# Point-of-care ultrasound (POCUS) pediatric resident training course: a cross-sectional survey

**DOI:** 10.1186/s13052-024-01652-7

**Published:** 2024-04-23

**Authors:** Manuela Lo Bianco, Santiago Presti, Maria Carla Finocchiaro, Gian Luca Trobia, Tiziana Virginia Sciacca, Maria Elena Cucuzza, Elia Caudullo, Giacomo Calcara, Martino Ruggieri, Vita Antonella Di Stefano

**Affiliations:** 1 Postgraduate Training Program in Pediatrics, Department of Clinical and Experimental Medicine, University of Catania, via S. Sofia, 78, 95125 Catania, Italy; 2 Pediatric Unit and Pediatric Emergency, Room of Emergency Hospital Cannizzaro, Via Messina 829, 95126 Catania, Italy; 3 U.O. Department of Diagnostic for Images, Emergency Hospital Cannizzaro, Via Messina 829, 95126 Catania, Italy; 4 U.O.S.D. Diagnostics for Emergency Imaging, Emergency Hospital Cannizzaro, Via Messina 829, 95126 Catania, Italy; 5 Unit of Clinical Pediatrics, Department of Clinical and Experimental Medicine, University of Catania, A.O.U. “Policlinico”, P.O. “G. Rodolico”, via S. Sofia, 78, 95125 Catania, Italy

**Keywords:** POCUS, Survey, Pediatric residents, Training course, Ultrasound

## Abstract

**Background:**

Point-of-care ultrasound (POCUS) is becoming increasingly crucial in the Pediatric Emergency Department for objective patient examination. However, despite its growing interest and wide-ranging applications, POCUS remains relatively unexplored in general pediatric training and education. Many physicians still find it challenging to comprehend and implement.

**Methods:**

A theoretical-practical POCUS course for pediatric residents was conducted at the University of Catania, Italy. The course’s effectiveness and practical impact on residents was assessed through a pre-post training survey. The first part of the questionnaire focused on the self-perceived time needed to learn how to recognize the following conditions using POCUS: (i) *Pleural effusion* (ii) *Lung consolidation* (iii) *Pneumothorax* (PNX) (iv) *Cardiac contractility* (v) *Pericardial effusion* (vi) *Perisplenic effusion* (vii) *Morison’s pouch effusion* (viii) *Douglas’ pouch effusion (ix) Filling and collapsibility of the inferior vena cava*. In the second part, we compared the potential role of POCUS in (i) *Reducing the use of ionizing radiation* in children (ii) *Increasing the sense of security in diagnosis and treatment* decisions making and (iii) *Increasing the residents’ confidence level* with POCUS after the course on a 1-to-10 rating scale.

**Results:**

Seventy-two residents participated in the study. The statistical analysis showed significant pre-post differences in almost all the items considered, except for *“cardiac contractility”* and *“PNX”.* Furthermore, the perceived potential role of POCUS in reducing ionizing radiation usage and the sense of security in diagnosis and treatment decisions showed statistically significant differences (*p* < 0.05) before and after the course. Data analysis also revealed a consistently high confidence level with POCUS after the course.

**Conclusions:**

The results highlight the importance of including a POCUS track course in pediatric post-graduate programs due to its simplicity, rapid learning time, and clinical usefulness. Based on these findings, it would be recommended to increase the teaching hours dedicated to the recognition of pneumothorax and cardiology POCUS examination. Emphasizing POCUS training in pediatric education can enhance patient care and diagnostic accuracy while minimizing radiation exposure.

**Supplementary Information:**

The online version contains supplementary material available at 10.1186/s13052-024-01652-7.

## Background

Point-of-care ultrasound (POCUS) has become an essential tool to examine patients in the Pediatric Emergency Department (PED) [1, 2]. It has numerous clinical applications, including assessing the hemodynamic state and respiratory distress, searching for conditions like pneumothorax (PNX), interstitial lung involvement, consolidation, atelectasis, and pleural effusion. POCUS is also valuable in abdominal scans for identifying intrabdominal fluids, aortic anomalies, and assessing hydronephrosis or bladder distention. Thus, in trauma cases, the focused assessment with sonography in trauma (FAST) protocol let to identify an intrabdominal sources of bleeding. Additionally, POCUS can assist in procedures like thoracentesis, paracentesis, airway assessment, dynamic cricothyroidotomy [3–8].

Since 2003, surveys on POCUS training in residency programs have emphasized the importance of incorporating bedside ultrasonography into training [9]. Recent studies have shown increased implementation of POCUS skills in various medical fields. However, when comparing pediatric subspecialties with their adult counterparts, POCUS remains relatively unexplored in general pediatric training and education [10]. Several physicians still find it challenging to understand and use this methodology, despite the advantage of easily examining children with ultrasound, especially using high-frequency probes that provide higher resolution images [10]. As a result, POCUS is not commonly included in pediatric clinical guidelines.

To address this gap, we developed a theoretical-practical POCUS course, attended by all residents in the Pediatrics post-training program at the University of Catania, Italy. The objective was to evaluate the course’s effectiveness and its practical impact on the residents. We administered a questionnaire before and after the POCUS training course to gather data on the residents’ perspectives and self-perceived benefits and confidence, providing valuable insights for improving pediatric training programs and promoting the use of POCUS in pediatric healthcare settings.

## Methods

Seventy-two pediatric residents from the University of Catania, Italy, voluntary participated in a POCUS training course in the academic year 2022/2023. None of the residents had previously undergone a POCUS course, and they had no prior ultrasound skills.

The course was organized by the Department of Pediatric and Pediatric Emergency Room at Cannizzaro Emergency Hospital, Catania, Italy. Medical trainees and residents from other specialties were not included in this study.

The training program spanned two days, with theoretical lessons in the mornings (4 h per day) and practical exercises in the afternoons (4 h per day), making the whole course last for 16 h. The course was conducted by six tutors who are experts in POCUS, each of whom held a diploma in Pediatric and Neonatal Ultrasound from the Italian Society of Ultrasonology in Medicine and Biology (SIUMB). They had undergone various professional training courses on POCUS and had more than 5 years of practical experience in the field. The tutors were divided into four stations and simultaneously supervised twelve learners. The ultrasound machines used during the course were Esaote MyLab™ฏX8 Platform. The topics of the course involved basic concepts of ultrasound, thorax ultrasonography, the use of POCUS applied in pediatric cardiovascular evaluation and the FAST protocol. Moreover, practical simulations were conducted using real cases from the pediatric emergency scenarios of the PED, (patients aged 0–16 years).

**Fig. 1:**
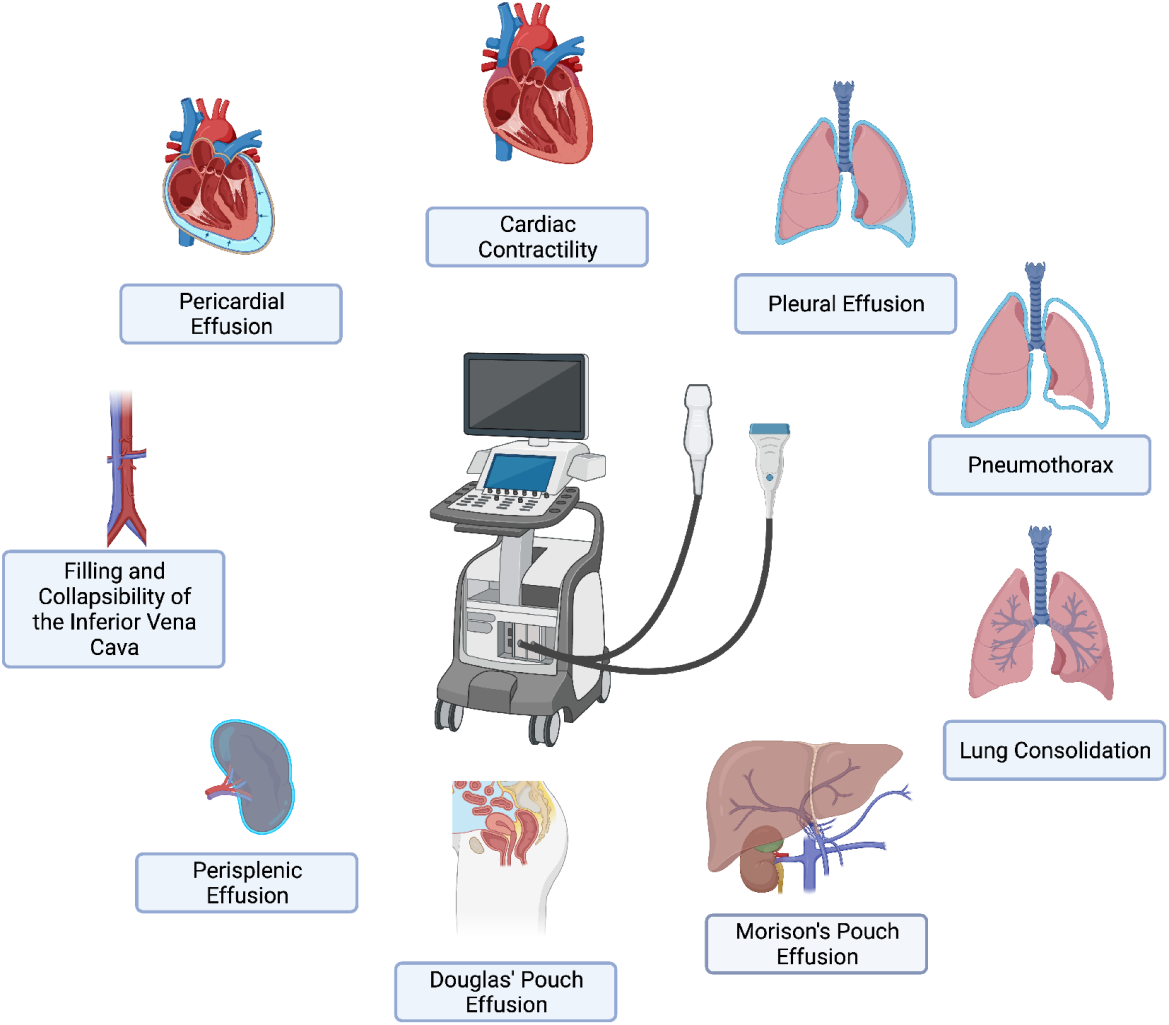
The figure illustrates the training self-assessment survey items, which have been based on the course curriculum

To assess the effectiveness of the course, a pre- and post-training survey was administered using Google Forms. The questionnaire used for the POCUS training course evaluation is available as Supplementary Material for this article. It aimed to collect data on the self-perceived time needed by each resident to learn how to recognize and obtain a correct diagnosis of the following conditions using POCUS: (i) *Pleural effusion* (ii) *Lung consolidation* (iii) *Pneumothorax* (PNX) (iv) *Cardiac contractility* (v) *Pericardial effusion* (vi) *Perisplenic effusion* (vii) *Morison’s pouch effusion* (viii) *Douglas’ pouch effusion (ix) Filling and collapsibility of the inferior vena cava*, (Fig. 1). After the completion of the survey, the authors categorised the answers as follow: < 2 h; 2-to-4 h; 4-to-6 h; 6-to-8 h; > 8 h.

In the second part of the questionnaire, participants were asked to rate the self-perceived role of POCUS training course in (i) *Reducing the use of ionizing radiation* in children (ii) *Increasing the sense of security in diagnosis and treatment* decisions-making (iii) *Increasing* residents’ *confidence level with the procedure.* Specifically, a 1-to-10 rating scale was used with 1 indicating “*not at all*” and 10 indicating “*extremely confident*”.

Therefore, the effectiveness of the course was assessed based on the self-reported decrease in time required to reach an accurate diagnosis, the reduction in the utilization of unnecessary imaging, particularly those involving ionizing radiation, and consequently, the decrease in needless specialist consultations.

The questionnaire was carried out on the basis of previously reported studies in the scientific literature. For this purpose, we selected and adapted items from published articles and chapters that outlined the most relevant information for pediatric POCUS [10–16].

The questionnaire was designed to be uniquely linkable for each participant, allowing for the application of paired statistical measures. Moreover, it was completely anonymous and the study was carried out in accordance to the ethical standards of the declaration of Helsinki. Volunteers signed informed consent and were informed to their rights.

### Statistical analysis

For statistical analysis, the software GraphPad version 6.00 (La Jolla, California, USA) was used. The dependent variables were analyzed with a 2-tailed paired t-test through the Wilcoxon test comparing the two groups, before and after the training. The assumption of normality was performed with the Shapiro-Wilk test. Alpha was set at 0.05.

## Results

A total of 72/72 pediatric residents participated in the POCUS training course, with a 100% response rate from the School of Pediatrics of the University of Catania, Italy.

**Fig. 2:**
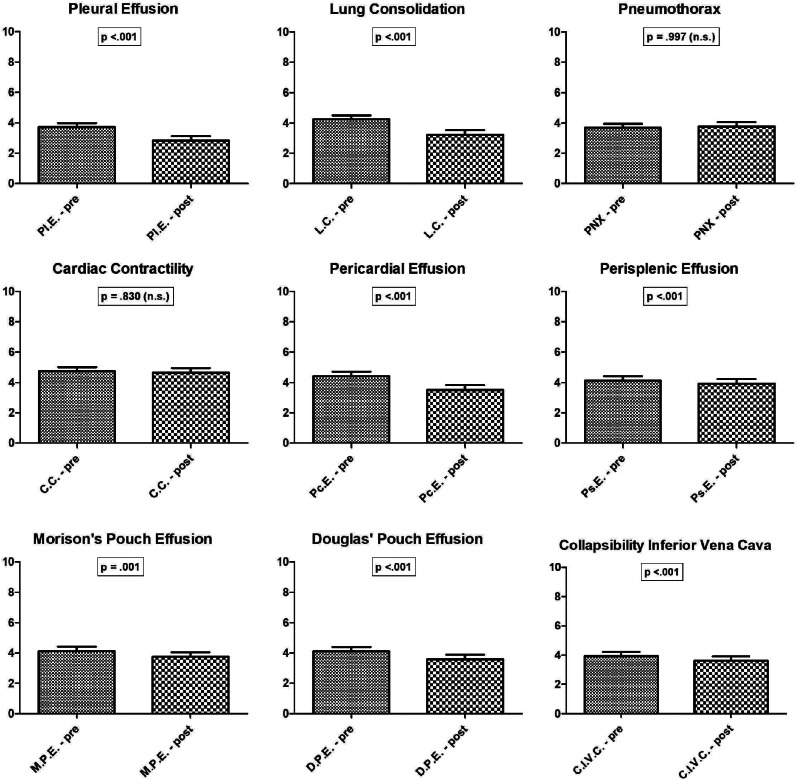
Statistically significant pre-post differences in perceived learning hours for point-of-care ultrasound skills: a survey-based analysis

The survey results revealed statistically significant pre-post differences (*p* < 0.05) in the number of hours considered necessary to learn POCUS skills, as perceived from participants, for the following items: *“Pleural effusion,” “Lung consolidation,” “Pericardial effusion,” “Perisplenic effusion,” “Morison’s pouch effusion,” “Douglas’ pouch effusion,” “Filling and collapsibility of the inferior vena cava”*. Conversely, for the items “*Cardiac contractility*” and “*Pneumothorax* (PNX)”, no statistically significant difference was found in the number of hours considered necessary before and after the course, (Fig. 2).

In addition, the survey results showed a statistically significant differences (*p* < 0.05) regarding the potential role of POCUS both in *Reducing the use of ionizing radiation* on children in residents’ clinical practice, and in their *Decision-making process concerning diagnosis and treatment* options.

**Fig. 3:**
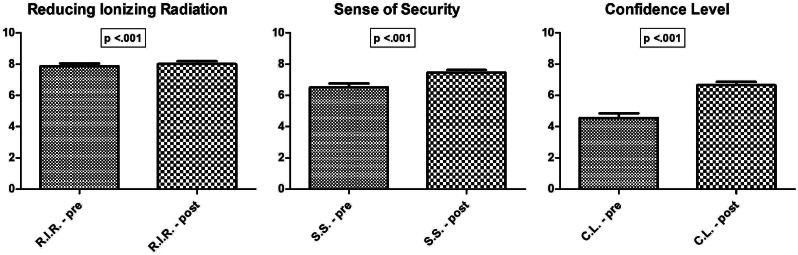
Statistically significant pre-post differences in perceived benefit for reducing ionizing radiation usage in children, increased sense of security in diagnosis and treatment decisions-making and increased residents’ confidence level with POCUS, using a 1-to-10 rating scale with 1 indicating “*not at all*” and 10 indicating “*extremely confident* “

Finally, participants’ responses indicated an overall high *Confidence level* and a positive impact with POCUS procedure, as shown in Fig. 3.

## Discussion

POCUS is an invaluable tool for pediatric specialists to enhance diagnostic accuracy. It is rapidly and easily learnt, even by medical students [17]. Therefore, it is crucial for pediatric residents to incorporate it into their routine clinical practice. For this reason, a theoretical-practical POCUS course was organized by expert tutors at the Cannizzaro Hospital of Catania, Italy. This residential course aimed to teach POCUS to all the residents attending the post-graduate programme in Pediatrics at the University of Catania, Italy, from the first to the fifth year of attendance.

The effectiveness of the course was assessed by a pre- and post-training survey, which was realized on the basis of previously reported studies. For instance, McGuinness et al. in their cross-sectional survey administered a questionnaire to assess the perceived usefulness and barriers of POCUS among faculty, residents, and graduates from a pediatric academic medical center. Similarly, Lewis et al. reported a questionnaire aimed to assess the need for introducing a POCUS curriculum to the internal medicine program. Furthermore, Kessler et al. investigated residents and students’ selection of the most useful applications of ultrasound in their future clinical practice [10–16].

The results from the questionnaire administered before and after the POCUS course indicated a statistically significant difference in the perceived number of hours necessary to learn most ultrasound skills by each resident, compared to the expected number of hours required before attending the course. This outcome suggests that pediatric residents have not only reduced their effort expectancy but also significantly increased their confidence in the POCUS technique after only a few hours of training. Consequently, the course’s effectiveness was evaluated based on the self-reported reduction in time needed to achieve an accurate diagnosis, diminished reliance on unnecessary imaging, especially those requiring ionizing radiation, and a subsequent decrease in unnecessary specialist consultations.

The only exceptions observed regarded the items “*Cardiac contractility*” and “*Pneumothorax*”: the number of hours considered suitable for recognition of these conditions was higher, although not statistically significant, than those perceived before attending the training. This result may suggest that the course probably had a less pronounced effect on the residents’ ability to learn these particular skills or, alternatively, they realized that these conditions themselves are especially complex. Furthermore, it may reflect a lack of knowledge in this field in pediatric settings, as it may be prerogative of cardiology/pneumology specialists, according to the residents’ opinions.

Nevertheless, POCUS should be considered a valuable tool to guide pediatricians, and overall physicians, in recognizing and differentiating several aetiologies of cardiac arrest, such as left ventricular dysfunction, hypovolemia, and tamponade, potentially included in the Advanced Cardiovascular Life Support (ACLS) algorithm [18]. Moreover, more training to recognize PNX would be also beneficial since it has been shown that the sensitivity and specificity of POCUS for its identification are comparable to traditional radiography, and sometimes even superior [19]. Thus, POCUS could reduce the quantity of administered radiations to children and it also enhances the shared understanding of patient diagnostics among providers and patients [20].

For these reasons, the above-mentioned topics should be deepened within the POCUS programme, devoting more hours to these topics within the programme and possibly involving specialists in the field, (e.g. cardiologists, pneumologists, radiologists).

A national survey of POCUS scholarly tracks in emergency medicine (EM) conducted by Alerhand et al. reported that only 28.6% of responding programs in the United States had a POCUS scholarly track, while the situation was different in Canada, where nearly all EM programs included POCUS training (though not universally standardized in every setting) [21, 22]. Wang et al. also found that the lack of educational opportunities was the main barrier to POCUS learning, as identified by the majority of surveyed Program Directors (PDs) (64%) [23]. Similarly, in the Italian pediatric residency programs, the teaching of POCUS is lacking and often limited to informal bedside teaching. In accordance with this issue, all the responding residents (72/72, 100%) in this study expressed the need for a POCUS course to be included in the post-graduate program and pediatric curricula. The importance of POCUS lies not only in its potential role in reducing the use of ionizing radiation in children but also in the increased level of confidence in making diagnostic and treatment decisions. However, it is essential to consider the costs of external POCUS courses for residents, which can be very expensive and consequently not affordable for everyone. Despite being highly recommended by various organizations worldwide, the incorporation of POCUS into official residency programs in Italy has not occurred, possibly due to the lack of national surveys addressing this topic [24–26].

## Limitations

The main limitation of this study is the relatively small sample size, as it only included pediatric residents from the University of Catania. Therefore, the findings may not be fully representative of the entire population of pediatric residents in Italy. However, the study can be considered a valuable pilot study for initiating a larger, national survey that includes a more diverse and broader sample of pediatric residents from various institutions across the country. A larger study is necessary to provide more robust and generalizable results regarding the perceptions and attitudes towards POCUS among pediatric residents in Italy.

A significant challenge of the study relies on a questionnaire that captures the personal perceptions and self-assessments of participants. Although this approach offers valuable insights into individual experiences, it may also introduce some subjectivity and bias. Notably, Italy lacks an officially approved questionnaire for POCUS, potentially affecting the standardization and comparability of findings. While the questionnaire successfully records the self-reported knowledge gains and residents’ perspectives, we acknowledge its limitations in assessing direct clinical impact and practical skills, highlighting a direction for future investigation and research, hopefully including a control group of residents who did not attended any POCUS course.

Despite these limitations, the study still holds significance as it sheds light on the current perceptions and attitudes towards POCUS among pediatric residents in a specific region. It highlights the need for further research on a national level to gain a more comprehensive understanding of the role and impact of POCUS in pediatric medical education in Italy. Additionally, the study underlines the importance of considering the formal implementation and standardization of POCUS training and assessment in pediatric residency programs to ensure a more objective and reliable evaluation of residents’ skills and competencies in using this valuable diagnostic tool.

## Conclusions

The results of this study suggest the significant impact of a theoretical-practical POCUS course on pediatric residents’ confidence levels in recognizing specifical conditions using POCUS. The course led to reduced self-perceived learning time for most ultrasound skills, indicating the rapid learning potential of POCUS. This finding indicates that pediatric residents have reduced their effort expectancy and improved their level of confidence with POCUS technique, in few hours of training.

Additionally, residents showed a greater awareness of the potential role of POCUS in reducing ionizing radiation usage and enhancing diagnostic and treatment decision-making.

Despite the positive outcomes, challenges remain, particularly regarding the recognition of more complex conditions such as “*Cardiac contractility*” and “*Pneumothorax*.” Further emphasis on these topics within the POCUS program, possibly with involvement from specialists, could enhance residents’ proficiency in these areas.

Given the high confidence levels reported by residents after the course, it would be recommended to include POCUS training as a regular track in pediatric post-graduate programs. This would ensure that future pediatricians are well-equipped with this valuable tool, enhancing patient care, diagnostic accuracy, and minimizing radiation exposure in pediatric healthcare settings.

National surveys and guidelines may help advocate for the integration of POCUS into official pediatric residency programs, ensuring a standardized and widespread approach to pediatric ultrasound education in Italy and beyond.

### Electronic supplementary material

Below is the link to the electronic supplementary material.

Supplementary Material 1

Supplementary Material 2

## Data Availability

The datasets used and/or analysed during the current study are available from the corresponding author on reasonable request.

## Abbreviations

POCUS: Point-of-care ultrasound
PNX: Pneumothorax
PED: Pediatric Emergency Department
FAST: focused assessment with sonography in trauma
SIUMB: Society of Ultrasonology in Medicine and Biology
ACLS: Advanced Cardiovascular Life Support
