# Supplementary material for: Point-of-care ultrasound (POCUS) pediatric resident training course: a cross-sectional survey
Source: Ital J Pediatr. 2024 Apr 23;50:82. doi: 10.1186/s13052-024-01652-7 (PMC11036774; doi:10.1186/s13052-024-01652-7)
Supplement: Supplementary file 1 — Supplementary Material 1 [file 13052_2024_1652_MOESM1_ESM.docx]

**POCUS course questionnaire**

| **Self-Perceived Time Needed to Learn POCUS Diagnostics** |
| --- |
|  |
| For each of the following conditions, please estimate the self-perceived time it takes you to learn how to recognize and obtain a correct diagnosis using POCUS: |
|  |
| - Pleural effusion |
| - Lung consolidation |
| - Pneumothorax (PNX) |
| - Cardiac contractility |
| - Pericardial effusion |
| - Perisplenic effusion |
| - Morison’s pouch effusion |
| - Douglas' pouch effusion |
| - Filling and collapsibility of the inferior vena cava |
|  |
| Please indicate the time required: |
| - Less than 2 hours |
| - 2 to 4 hours |
| - 4 to 6 hours |
| - 6 to 8 hours |
| - More than 8 hours |

| **Impact of POCUS Training** On a scale from 1 to 10, with 1 being "not at all" and 10 being "extremely confident," please rate the role of the POCUS training course in: |
| --- |
|  |
| - Reducing the use of ionizing radiation in children   1 2 3 4 5 6 7 8 9 10 |
| - Increasing the sense of security in diagnosis and treatment decision-making   1 2 3 4 5 6 7 8 9 10 |
| - Increasing your confidence level with POCUS   1 2 3 4 5 6 7 8 9 10 |
|  |
| **Additional Feedback** Is there anything that could be improved to enhance the effectiveness of the course? |
