# Supplementary material for: Point-of-care ultrasound (POCUS) pediatric resident training course: a cross-sectional survey
Source: Ital J Pediatr. 2024 Apr 23;50:82. doi: 10.1186/s13052-024-01652-7 (PMC11036774; doi:10.1186/s13052-024-01652-7)
Supplement: Supplementary file 2 — Supplementary Material 2 [file 13052_2024_1652_MOESM2_ESM.docx]

To the Editor-in-Chief

Italian Journal of Pediatrics

Prof. Giovanni Corsello, MD

*University of Palermo, Palermo, Italy*

**Subject**: Submission of Manuscript – *“Point-of-care ultrasound (POCUS) pediatric resident training course: a cross-sectional survey”.*

Dear Prof. G. Corsello,

I hope this letter finds you well. I am writing to submit our manuscript entitled *“Point-of-care ultrasound (POCUS) pediatric resident training course: a cross-sectional survey”* for consideration for publication in the Italian Journal of Pediatrics.

Our manuscript addresses a critical gap in pediatric training programs, focusing on the implementation and effectiveness of a Point-of-Care Ultrasound (POCUS) course for pediatric residents at the University of Catania, Italy. The study explores the impact of this course on residents' confidence levels, self-perceived learning time, and awareness of POCUS applications. This research is timely and relevant, considering the increasing importance of POCUS in pediatric emergency care and the existing gap in formal training programs.

We believe that the findings presented in our manuscript contribute significantly to the field of pediatric medical education. This pilot-study not only highlights the positive impact of the POCUS course on reducing learning time and increasing confidence but also identifies specific areas that may require further emphasis in future training programs, such as the recognition of complex cardiac conditions and pneumothorax.

Moreover, our study underlines the current lack of formal POCUS training within pediatric residency programs in Italy, emphasizing the need for a standardized approach to ensure that future pediatricians are well-equipped with this valuable diagnostic tool.

We confirm that all authors have approved the manuscript for submission, and the content has not been published or submitted elsewhere. There are no conflicts of interest to declare.

We believe that our manuscript aligns well with the mission and scope of the Italian Journal of Pediatrics, and its publication would contribute to the ongoing dialogue on improving pediatric medical education. We appreciate your time and consideration of our submission.

Thank you for your attention, and we look forward to the opportunity of sharing our work with the readership of the Italian Journal of Pediatrics.

Sincerely,

Manuela Lo Bianco, MD

Postgraduate Training Program in Pediatrics, Department of Clinical and Experimental Medicine, University of Catania, via S. Sofia, 78, 95125, Catania, Italy.

lobianco.manuela@gmail.com
